# Regimens and Response Assessment in Minimally Invasive Image-Guided Therapies for Vascular Malformations: Insights from a Large Cohort Study at a Tertiary-Care Hospital

**DOI:** 10.3390/life14101270

**Published:** 2024-10-05

**Authors:** Gesa Doreen Savic, Giovanni F. Torsello, Anne Frisch, Gero Wieners, Uli Fehrenbach, Timo Alexander Auer, Willie Magnus Lüdemann, Bernhard Gebauer, Lynn Jeanette Savic

**Affiliations:** 1 Department of Radiology, Campus Virchow-Klinikum, Charité-Universitätsmedizin Berlin, Freie Universität Berlin and Humboldt-Universität zu Berlin, Augustenburger Platz 1, 13353 Berlin, Germany; gesa-doreen.savic@charite.de (G.D.S.); anne.frisch@charite.de (A.F.); uli.fehrenbach@charite.de (U.F.); timo-alexander.auer@charite.de (T.A.A.); willie-magnus.luedemann@charite.de (W.M.L.); bernhard.gebauer@charite.de (B.G.); 2 Department of Diagnostic and Interventional Radiology, Universitätsklinikum Göttingen, 37075 Göttingen, Germany; giovanni.torsello@med.uni-goettingen.de; 3 Department of Radiology, Carl-Thiem-Klinikum Cottbus, 03048 Cottbus, Germany; g.wieners@ctk.de; 4 Berlin Institute of Health, Charité—Universitätsmedizin Berlin, 10117 Berlin, Germany; 5 Experimental Clinical Research Center (ECRC), Charité—Universitätsmedizin Berlin and Max-Delbrück-Centrum für Molekulare Medizin (MDC), 13125 Berlin, Germany

**Keywords:** vascular malformations, sclerotherapy, venous malformations, minimally invasive therapies, magnetic resonance imaging

## Abstract

This retrospective study was aimed at characterizing vascular malformations (VMFs) presenting for minimally invasive image-guided therapies (MIT) at a tertiary-care center and evaluating treatment regimens and image-based outcomes using MRI. We analyzed demographic, disease-related, and radiologic features of VMFs presenting to interventional radiology between May 2008 and August 2020 using compendium vascular anomaly (Compva) criteria. MIT and specific agents were evaluated, and treatment effects were assessed through volumetry and mean signal intensity (MSI) on multiparametric longitudinal MRI. The statistics included the paired *t*-test, ANOVA, and Fisher’s exact test. The cohort included 217 patients (mean age 30 ± 18.4 years; 134 female). Venous malformations were most common (47%). VMFs were frequently located in the head-neck region (23.5%), legs (23.04%), and arms (13.8%). Among 112 treatments, sclerotherapy was performed most frequently (63.9%), followed by embolization (19.3%). MRI showed a significant reduction in T2 MSI for venous (1107.95 vs. 465.26; *p* = 0.028) and decreased contrast media uptake for lymphatic malformations (557.33 vs. 285.33; *p* = 0.029) after sclerotherapy, while the lesion volumes did not change significantly (*p* = 0.8). These findings propose MRI-derived MSI as a potential non-invasive biomarker for assessing the response of VMF to MIT. By leveraging MRI, this study addresses challenges in managing rare diseases like VMFs, while advocating for standardized approaches and prospective studies to better link imaging findings with clinical outcomes.

## 1. Introduction

Vascular malformations (VMFs) belong to the group of congenital vascular anomalies along with vascular tumors. They arise from spontaneous mutations during embryogenesis [1]. While they exist since birth, they never regress spontaneously but are frequently observed to grow and progress over time, especially as a result of growth spurts and/or hormonal changes [2,3], and become noticeable through unspecific symptoms such as functional limitations or pain [4,5].

The International Society for the Study of Vascular Anomalies (ISSVA) categorizes VMFs as simple, combined, VMFs of major named vessels, and those associated with other anomalies [6]. The simple malformations are further divided into low-flow (capillary, lymphatic, and venous) and fast-flow (arteriovenous and arteriovenous fistulas) malformations. The most common types are low-flow venous malformations (VMs) accounting for approximately 50% of all VMFs, followed by lymphatic malformations (LMs) [5,7]. 

Treatment of VMFs is mainly pursued when patients show relevant impairment. Upon failure of conservative, non-invasive treatments such as compression or physiotherapy, minimally invasive therapies (MIT) including image-guided sclerotherapy or embolization are a mainstay therapy [5]. However, regimens remain non-standardized with a variety of agents being used for these treatments.

VMFs are rare diseases with a very heterogeneous presentation and extension, making it difficult to perform a large prospective study with comparable patient cases. Thus, there are only limited data on the therapeutic success of MIT in relation to the type and size of the malformation. In addition, little evidence exists on symptom changes and complication rates or methods for measuring lesion response to therapy. 

Therefore, this study aims to characterize the cohort of VMF patients presenting for MIT therapy at a tertiary-care center, systematically analyze treatment protocols, and evaluate therapeutic efficacy on MRI. 

## 2. Materials and Methods

### 2.1. Study Cohort and Design

This retrospective single-center study was approved by the institutional review board and adheres to the Declaration of Helsinki. Informed consent was waived due to the retrospective design. Consecutive patients presenting with any form of VMF for an initial consultation regarding MIT at the Department of Radiology at a tertiary-care center between May 2008 and August 2020 were included in the analysis. Clinical and imaging data from all patients were reviewed and analyzed to determine: (a) the patient population characteristics; (b) the symptoms; (c) the types, localizations, and imaging features of VMFs; (d) the therapeutic strategies; and (e) the imaging-based response to MITs. 

### 2.2. Medical Record Review and Minimally Invasive Therapies

Demographics, information on the type and location of malformation, and clinical symptoms were extracted from patient records. In addition to the patient files, MR images and reports were evaluated to confirm the localization and extent of the malformations. Additionally, angiography imaging and reports were reviewed to collect data on the type of therapy and specific materials used for sclerotherapy or embolization. 

### 2.3. MRI Acquisition and Image Analysis 

All available MR imaging data obtained prior to and after therapy were analyzed. MRI was acquired on 1.5 or 3T scanners (Siemens Healthineers AG, Forchheim, Germany; Philips, Hamburg, Germany; GE Healthcare, Düsseldorf, Germany; Canon Inc., Tokyo, Japan), and the protocols included T1-weighted sequences with and without gadolinium-based contrast agents (Gadovist, Bayer Vital GmbH, Leverkusen, Germany; Dotarem, Guerbet, Sulzbach, Germany; Cyclolux, Sanochemia Pharmazeutika GmbH, Neufeld an der Leitha, Austria; Magnevist, Bayer Vital GmbH; OptiMARK, Medtronic GmbH, Meerbusch, Deutschland) and T2-weighted sequences. 

#### 2.3.1. Volumetry

In patients with more than one therapy session, the first recorded treatment during the observation period was analyzed with regards to the modality and reagents used, and the imaging prior to this therapy was considered the baseline imaging. First, the extent of the malformations was determined in three dimensions on axial and coronal images using a caliper tool and the formula length x width x height to calculate the lesion volume pre- and post-therapy. Volumetry was performed on the sequence where the lesion could be best distinguished. Secondly, the sequences were examined for MRI-specific characteristics of the VMF to determine the character and type of VMF based on established criteria by the Compendium Vascular Anomalies [8] (see Table S1 and Figure S1). Based on the MRI parameters and the therapeutic agents used during the intervention, the type of VMF could be determined and reconciled with the report of the care center. The VMFs were classified as VM, LM, venous-lymphatic malformation (VLM), arteriovenous malformation (AVM), or others (e.g., hemangioma; excluded from the statistical analysis).

#### 2.3.2. Mean Signal Intensities (MSI)

In addition, MSI was assessed on T2-, native T1-, and contrast-enhanced T1-sequences, respectively. Briefly, three reference regions of interest (ROIs) of 1 cm^3^ were placed in representative areas of the VMF and outside the VMF in adjacent, unaffected localizations of the same tissue or organ in each available sequence. In VMFs with sedimentation or flow-voids, the ROIs were placed outside of these areas in more homogenous parts of the lesion. The mean MSI was calculated and compared longitudinally (baseline vs. follow-up 1 vs. follow-up 2). Additionally, the ratios of MSI measured within and outside of the lesion were calculated and evaluated over time. To determine the imaging-based biomarkers of successful treatment, post-therapeutic changes of the lesion volume and MSI were assessed. The image analysis was performed by a PhD-student and two radiologists in consensus readings with two and four years of experience in interventional radiology. 

### 2.4. Statistical Analysis

Descriptive statistics were used to evaluate the patient data and VMF characteristics. Longitudinal changes of lesion volume or MSI were evaluated using a paired t-test (2 time points) or repeated measurement ANOVA (>2 time points). Additionally, patients were stratified according to baseline characteristics (i.e., sex, age), and sub-group analyses were performed using an unpaired *t*-test (2 groups) or ANOVA (>2 groups) or Fisher’s exact test. Statistical analyses were conducted using IBM SPSS Statistics (v29.0.0.0). *p*-values < 0.05 were considered statistically significant.

## 3. Results

### 3.1. Study Cohort and Patient Characteristics

By reviewing consecutive patient data, a total of 217 patients were identified who had presented with suspected VMFs to the Department of Radiology. The cohort consisted of 78 males (35.9%), 134 females (61.8%), and 5 individuals (2.3%) with unknown sex (only incomplete documentation available). The mean age at baseline was 28.01 ± 17.84 years in female patients and 32.63 ± 19.14 years in male patients (*p* = 0.088), respectively (see Table 1). 

**Figure 1:**
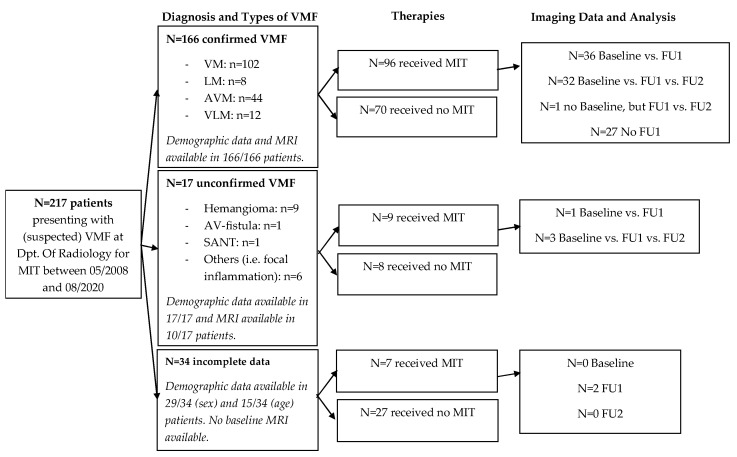
Composition of the cohort, divided into types, therapy, and availability of imaging data.

Date of birth or information on the patient’s age were available for 193 of 217 patients. Of all the patients, 39 patients (17.9%) had received a previous MIT of their VMF before the observation period for this study had started (Figure 1).

### 3.2. Types and Localization of VMFs

The prevalence of the types of VMF according to the Compendium Vascular Anomalies [8] in the entire cohort as well as stratified by sex is summarized in Table 1. VM were the most common type of VMF (102/217, 47%) in both female and male patients. No significant differences were observed regarding the types of VMF between female and male patients. 

Table 2 shows the localizations of VMFs in this cohort. The location was available for 213/217 patients (98.2%). The head-neck region (including the head-neck, eye, and jaw) was most affected by VMFs (n = 51, 23.5%), followed by the lower extremities (n = 50, 23.04%), and the upper extremities (n = 30, 13.8%).

### 3.3. Minimally Invasive Therapies 

A total of 112 patients (51.6%) received MITs for their VMF during the observation period. Of the 112 patients who received therapy, the type and specific agents used during the intervention were specified in 105 patients (96.3%) (summarized in Table S2). Both low- (n = 50, 79.9%) and high-flow malformations were most frequently treated with sclerotherapy (n = 13, 41.9%) (see Table 3). Three patients underwent surgery immediately after MIT. Patients who underwent standalone surgery (without prior MIT) were included in the study cohort of 217 patients but excluded from the therapeutic regimens list in Table 3 and from the image analysis (refer to Figure 1, “no MIT”).

### 3.4. Imaging-Based Response Assessment

Of all the 217 patients, 192 (88.5%) had received an MRI at baseline, but only 175 of those (91.2%) could be used for the evaluation of the volumetric data (e.g., due to insufficient image quality or incomplete datasets). Thirty-two of the treated patients with a confirmed VMF (33.3%) had a baseline, follow-up 1 and 2 MRI. Another 36 (37.5%) patients had a follow-up 1 MRI to compare to their baseline MRI. The median time between the baseline and the follow-up 1 MRI was 238 days (Interquartile range (IQR), 214.5 days). The median time between follow-up 1 and 2 was 256 days (IQR, 200.25 days).

#### 3.4.1. VMF Volumes

At baseline, the mean volume ± SD of all VMFs was 185.11 ± 508.9 cm^3^. The mean volume of all VMFs undergoing therapy was 140.42 ± 208.82 cm^3^. Overall, no significant change in lesion volume was observed from baseline to first follow-up (136.65 ± 208.04 cm^3^; *p* = 0.4), independent of the type of VMF. Similarly, no significant changes in lesion volumes were observed from baseline to follow-up 2 (144.23 ± 216.84 cm^3^; *p* = 0.06) or from follow-up 1 to follow-up 2 (*p* = 0.21). 

For the assessment of the treatment success of sclerotherapy as monotherapy, 48 of 63 patients (76.2%) with a baseline MRI and follow-up MRI were available. As for volumetry, 16 of 48 patients (33.33%) showed an enlargement of the VMF at the first follow-up, whereas a size reduction was observed in 32 patients (66.66%; 108,969.2 ± 168,325.5 mm^3^ vs. 107,552.16 ± 185,236.3 mm^3^; *p* = 0.797). 

**Figure 2:**
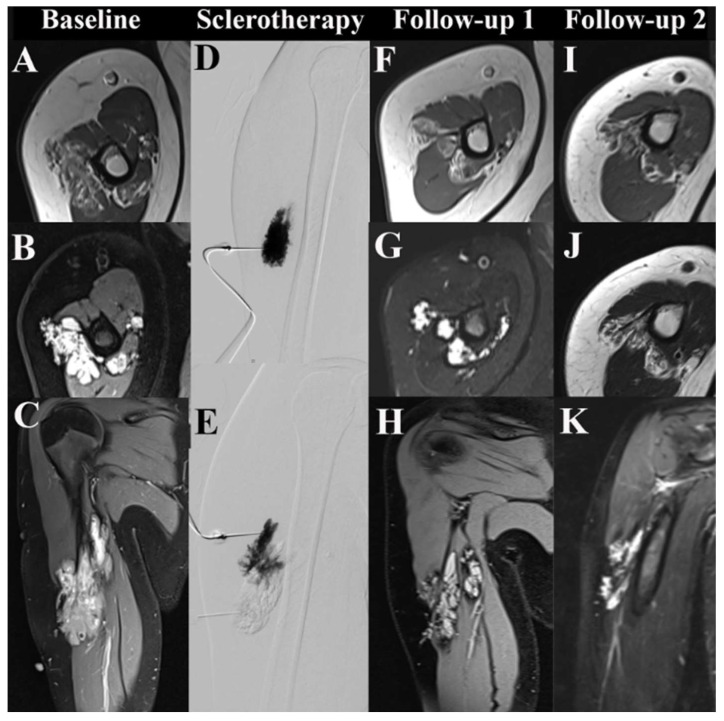
MRI of a low-flow venous malformation (VM) in the right upper arm before and after sclerotherapy. From top to bottom, axial T1-weighted sequences, axial T2-weighted sequences, and parasagittal contrast-enhanced sequences are shown in the first, second, and third rows. (**A**–**C**) show the baseline MRI of an untreated VM. (**D**,**E**) depict fluoroscopic images from ultrasound-guided percutaneous sclerotherapy using two direct punctures. (**F**–**H**) show MRI scans from follow-up 1 at seven months post-sclerotherapy. (**I**–**K**) depict MRI scans from follow-up 2 of the VM at 3.6 years post-sclerotherapy.

Nineteen patients with embolization had a baseline MRI and 13 (61.91%) also had a follow-up MRI. As for volumetry, six patients (46.15%) had increasing and seven (53.85%) had decreasing lesion volumes. However, no significant change in volumes became apparent (113,460.2 ± 198,258.9 mm^3^ vs. 143,475 ± 193,204.4 mm^3^; *p* = 0.095) (see Table 4 and Table 5, and Figure 2). 

#### 3.4.2. MSI Analysis

At baseline, the mean ± SD MSI of all lesions on the T2-weighted MRI was 777.4 ± 1208.72, on the T1-weighted MRI it was 558.03 ± 687.27, and on the contrast-enhanced T1-weighted MRI it was 718.86 ± 948.24. 

For the high-flow lesions, the MSI was 507.76 ± 341.23 on the T2-weighted MRI, 451.52 ± 400.25 on the T1-weighted MRI, and 699.44 ± 683.91 on the contrast-enhanced T1-weighted MRI sequences.

For the low-flow lesions, the MSI was 815.18 ± 1048.9 on the T2-weighted MRI, 606.36 ± 785.6 on the T1-weighted MRI, and 737.1 ± 1067.5 on the contrast-enhanced T1-weighted MRI.

**Figure 3:**
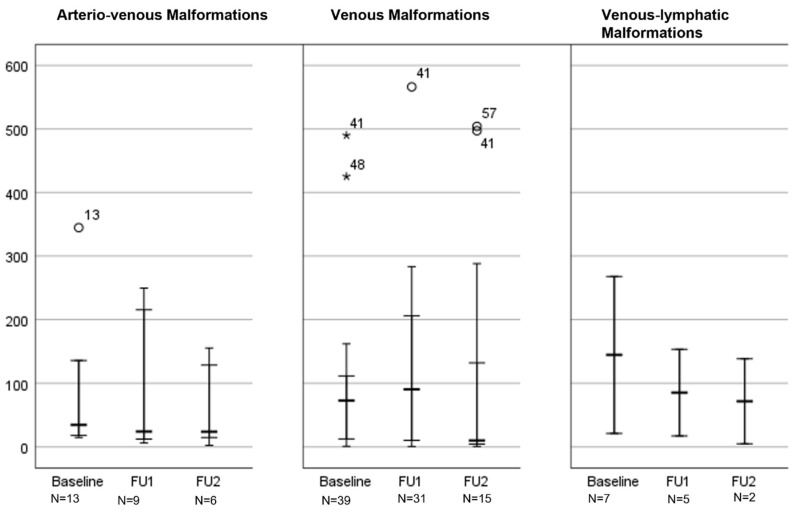
Changes in lesion volume after sclerotherapy sorted by type of vascular malformation. The box plot shows the type of malformation and the measured volumes at three time points before and after minimally invasive therapy. The mean, minimum, and maximum are indicated by the horizontal lines. Abbreviations: FU1 Follow-Up 1; FU2 Follow-Up 2.

**Figure 4:**
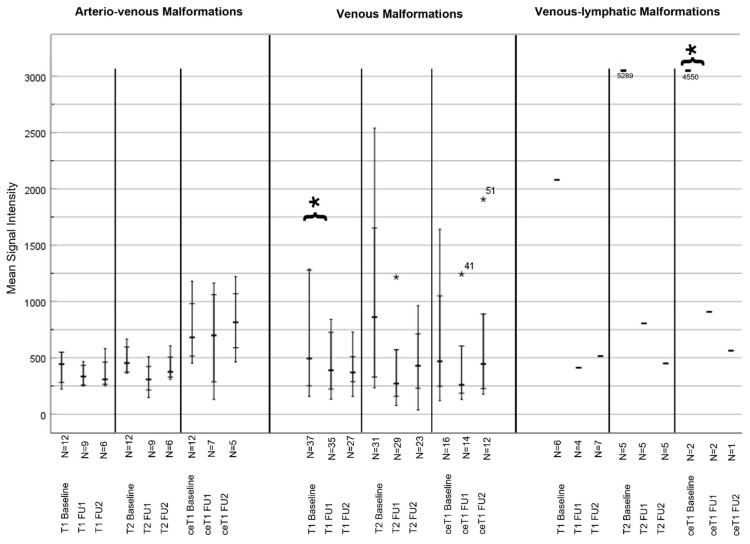
Change in the mean signal intensity after sclerotherapy sorted by type of vascular malformation. The changes in mean signal intensity are stratified by the type of malformation and the type of MRI sequence for 3 measuring points (baseline, follow-up 1 and 2). The mean, minimum, and maximum are indicated by the horizontal lines. * = *p* < 0.05 Abbreviations: FU1 Follow-Up 1; FU2 Follow-Up 2; ce contrast enhanced.

After sclerotherapy, a significant change in MSI from baseline to follow-up 1 was observed on the T2-weighted MRI in the VM group (1007.9 vs. 465.26; *p* = 0.028), as well as in the contrast-enhanced T1-sequences in the LM group (557.33 vs. 285.33; *p* = 0.029). However, postprocedural changes on native (804.56 vs. 577.48; *p* = 0.189) or contrast-enhanced T1-weighted sequences (605.13 vs. 468.13; *p* = 0.278) in the rest of the cohort were not statistically significant. A significant change was observed in the MSI ratios on the contrast-enhanced T1-sequence in the VM group (3.04 vs. 2.5; *p* = 0.011) (see Table 4 and Table 5, and Figure 3 and Figure 4).

### 3.5. Symptoms and Complications

Clinical symptoms prior to therapy were documented for 156 of 217 patients (71.89%). The most common symptom pre-therapy was pain (n = 112, 71.8%) followed by swelling (n = 72, 46.2%), and pressure sensations (n = 36, 23.1%).

After treatment, there were only 36 patients (16.6%) with available reported symptoms. Due to the limited availability of post-therapeutic clinical data, these data were summarized only as symptom improvement or worsening. Twenty (55.56%) patients reported deterioration or more than one remaining symptom. Thirteen (65%) of them had a low-flow and 7 (35%) a fast-flow malformation, respectively. On the other hand, 16 patients (44.44%) reported an improvement in symptoms; ten of those (62.5%) had a low-flow and six (37.5%) a fast-flow malformation (Table S3). Given the scarcity of the data, no statistical comparison was performed. During the observation period, additional MIT were performed to treat the remaining symptoms in 43 patients using sclerotherapy (n = 25, 58.1%), embolization (n = 7, 16.3%), a combination of sclerotherapy and embolization (n = 7, 16.3%), and other combinations (n = 4, 9.3%). 

## 4. Discussion

This large cohort study provides a comprehensive overview of the demographics, disease characteristics, treatment regimens, and imaging outcomes of 217 patients with VMF, with 112 undergoing minimally invasive therapy including sclerotherapy (63.9%) and embolization (19.3%). As a main finding, reduced signal intensities on the postprocedural T2-weighted MRI of VM or contrast-enhanced MRI of LM were revealed as surrogate markers of image-based response to sclerotherapy. Such tools could inform the management of patients with VMFs and guide further treatment planning in a currently non-standardized setting with various available treatment options. 

We applied established imaging criteria to classify VMFs on radiological imaging [8]. This can help avoid misdiagnosis and enable personalized treatment planning, particularly in rare diseases like VMF. In the existing literature, the most common VMFs are VMs representing a type of low-flow malformation, and VMs located in the head-neck region [9,10,11], which is consistent with observations in this cohort (102/217 VM; 51/217 lesion site in the head-neck region). Furthermore, in this large single-center cohort, the male:female-ratio was 1:1.72, confirming a higher incidence of VMFs in female patients [9]. 

To determine the best treatment for VMFs based on individual patient needs, a multidisciplinary approach involving vascular surgeons and interventional radiologists is essential. The choice between sclerotherapy and embolization or combinations depends on several factors, including the type, size, and location of the malformation, as well as the risk of complications, the patient’s anesthetic risk, and patient preferences. Sclerotherapy is typically used for slow-flow malformations like VMs, while embolization is more common for fast-flow malformations like AVM. Accordingly, the predominant therapy for low-flow malformations in this cohort was sclerotherapy (n = 50, 40.1%). In percutaneous sclerotherapy, the VMF is targeted under ultrasound and fluoroscopic guidance [12]. Commonly used agents cause an inflammatory reaction of the endothelium, adhesion of vessel walls, and ultimately shrinkage of the lesion [3,8,13,14]. However, this reaction takes weeks to months, and the VMF usually decreases its size but does not fully resolve. 

Electrosclerotherapy is a rather recent development of traditional sclerotherapy using needle electrodes to deliver electric shocks to the lesion, making the endothelium more permeable to the injected agent, mostly bleomycin. Both treatments are suitable for low-flow malformations, but electrosclerotherapy was introduced at the end of the observation period for this study and, thus, only accounts for a few cases [8,15,16].

Embolization is another option for fast-flow malformation, used to occlude the pathological vessels supplying the VMF. The technique requires catheter access to apply embolic materials through the feeding artery, including coils, plugs, or liquid embolics such as glue [8,17]. In this cohort, the patients with fast-flow malformations were mainly treated with sclerotherapy [8,17]. 

No therapy ensures complete resolution of the VMF but it oftentimes achieves size reductions, resulting in symptom improvement [8]. MIT offer several advantages over traditional surgery when treating VMFs, as they generally result in less trauma and fewer complications, reducing risks such as infection, bleeding, and damage to surrounding tissues. Unlike surgery, which can involve removing significant amounts of tissue, MIT allow for targeted therapy, preserving more of the healthy surrounding anatomy [18,19]. This precision also makes them particularly effective for VMFs in challenging or hard-to-reach areas of the body or VMFs that are difficult to separate from adjacent tissue. As a result, patients experience quicker recovery times, shorter hospital stays, and less postoperative discomfort. Besides the maintenance of function, MIT also tend to result in better cosmetic outcomes, as they avoid the scars and disfigurement that can accompany surgical procedures. Additionally, MIT can be repeated, if necessary, unlike surgery, which becomes more complex with each subsequent operation due to scar tissue and altered anatomy [18,19].

A large review reported on a few cases of skin damage and paresthesia after MIT [19]. In this cohort, only a few cases of ulceration and necrosis were recorded but no grading could be reliably assessed due to the retrospective character of the study. However, symptom reports were only available in a small subset of patients, with pain being the main symptom pre- and post-therapy. 

Alterations of signal intensities on MRI can be used to assess treatment response. In T2-weighted MRI, where liquids are best visualized, low-flow malformations appear hyperintense compared to the surrounding tissue due to the prolonged T2-relaxation time. A decreased MSI on a T2-weighted MRI after therapy may indicate that malformations are less fluid-filled with reduced perfusion. The image analysis revealed reduced signal intensity on the T2-weighted MRI after sclerotherapy, potentially serving as a non-invasive biomarker of treatment response. This reduction was particularly evident in VM (*p* = 0.028), though the volume of the VM had not changed significantly. Such imaging tools could indicate potentially undertreated lesions or areas of VMF for additional therapeutic sessions.

This finding further suggests that sclerotherapy is an effective MIT option for VM, as blood flow is reduced after the first treatment session, and only 42 patients (37.5%) required additional treatments during the observation period. Several studies investigated T2-weighted MRI for diagnosing and assessing the response in VMF. Specifically for the diagnosis of low-flow malformations, a T2-weighted MRI is recommended [8,20,21,22]. However, only scarce evidence supports the correlation of imaging changes with symptom improvement following MIT [23]. In this retrospective study, we could not correlate the decrease in signal intensity with symptom relief, as symptoms were reported at follow-up only in a small number of patients.

Surprisingly, significant differences in imaging after MIT were also observed in LM, which usually present with a diffuse microcystic phenotype or marginal contrast uptake prior to treatment [8]. Shrinkage was observed in about half of the VMFs after treatment in this study. Outliers with large malformations that could not be sufficiently treated in one session may have affected these results. Temporary hematomas after treatment, hardly distinguishable from VMFs, may have influenced size measurement. Thus, size or volume reduction should be measured once hematomas have healed.

This study has several limitations. It included a long observation period with new techniques introduced over time. However, this study was designed to represent real-world data and summarize experience in a rare disease, which requires longer observation periods to acquire sufficient and robust data for statistical analysis. Due to the retrospective nature of the study, only limited information on symptoms or complications were available, and the imaging data were incomplete for some patients and obtained at various time intervals. A subset of patients (n = 39) had undergone prior therapies before the study observation period. Due to the limited information available and their re-presentation with symptoms, they were not excluded from the image analysis. Additionally, variations of MRI scanners and imaging protocols may affect the image analysis, but we only chose very common sequences for analysis that should be reproducible independent of the MRI hard- or software. Furthermore, there is no consistency in the techniques or agents used for treatments, including 14 different substances, which make a direct comparison of treatment groups challenging. Therefore, no statistical tests were performed for symptom improvements to avoid statistical over- or underestimation. However, no complications were recorded that resulted in further imaging, treatment, or hospitalization after MIT. Lastly, VMFs often appear as combined malformations. In this study, they were classified according to the dominant characteristics and assigned to one type, which was also authoritative for the treatment regimen.

## 5. Conclusions

In conclusion, this large cohort study provides insights into the demographics and disease characteristics of 217 patients with VMFs, with 112 undergoing MIT including sclerotherapy (63.9%) and embolization (19.3%). The variety of treatments and combinations of therapeutic agents used underscores the need for standardized approaches and prospective studies to better correlate imaging with clinical outcomes. While the findings on the types and localization of VMFs in this cohort align with previous smaller case series, this study introduces MRI-derived MSI as a promising non-invasive biomarker for evaluating the response of VMFs to MIT. Specifically, reduced T2 signal intensities in venous and decreased contrast media uptake in lymphatic malformations post-MIT suggest that MRI could play a critical role in guiding treatment and improving patient monitoring for more personalized therapies in this rare disease. 

## Figures and Tables

**Table 1:** Distribution of age and type of malformation stratified by sex.

	All	Female	Male	N/A	*p*-Value
**N (%)**	217 (100%)	134 (61.8%)	78 (35.9%)	5 (2.3%)	
**Age ø in years**	29.68 ± 18.40	28.01 ± 17.84	32.63 ± 19.14		0.088
**Type of Malformation**					
**AVM**	44 (20.3%)	31 (70.5%)	13 (29.5%)		0.173
**VM**	102 (47%)	67 (65.7%)	35 (34.3%)		0.282
**VLM**	12 (5.5%)	7 (58.3%)	5 (41.7%)		0.469
**LM**	8 (3.7%)	3 (37.5%)	5 (62.5%)		0.124
**Others**	17 (7.8%)	8 (47.1%)	9 (52.9%)		0.150
**N/A**	34 (15.7%)	18 (52.9%)	11 (32.4%)	5 (14.7%)	

*p*-values indicate differences in age and prevalence of malformations between female and male patients, respectively. Abbreviations: AVM: Arterio-Venous Malformation VM: Venous Malformation VLM: Venous-Lymphatic Malformation LM: Lymphatic Malformation Others comprise hemangiomas, av-fistulas, SANT, others i.e., focal inflammations). N/A: incomplete data.

**Table 2:** Localization of vascular malformations in the entire cohort.

	Head Neck Mouth Tongue	Eye	Upper/ Lower Jaw	Shoulder	Arm	Hand	Thorax	Abdomen Pelvis Visceral Organs	Back Sacrum Flank	Genitals Groin Urinary Tract	Buttock	Upper Leg	Lower Leg	Knee	Foot	Bone	Multi- Focal	Unknown
**AVM**	8 (22.22%)	2 (28.6%)	2 (25%)	1 (10%)	3 (15%)	3 (33.3%)		2 (20%)	1 (12.5%)	5 (41.7%)	2 (40%)	7 (24.1%)	3 (14.3%)	1 (7.1%)	3 (23.1%)		1 (33.3%)	
**VM**	13 (36.11%)	4 (57.2%)	2 (25%)	8 (80%)	12 (60%)	5 (55.6%)	2 (33.3%)	2 (20%)	4 (50%)	4 (33.3%)	1 (20%)	14 (48.3%)	12 (57.1%)	11 (78.6%)	6 (46.2%)	1 (50%)	1 (33.3%)	
**VLM**	5 (13.88%)	1 (14.2%)	2 (25%)							1 (8.3%)			1 (4.8%)		1 (7.7%)	1 (50%)		
**LM**	2 (5.55%)		1 (12.5%)				1 (16.7%)		1 (12.5%)		2 (40%)	1 (3.5%)						
**Others**	1 (2.8%)			1 (10%)	1 (5%)		2 (33.3%)	4 (40%)	2 (25%)	1 (8.3%)			1 (4.8%)	2 (14.3%)	1 (7.7%)			
**No data**	7 (19.44%)		1 (12.5%)		4 (20%)	1 (11.1%)	1 (16.7%)	2 (20%)		1 (8.3%)		7 (24.1%)	4 (19%)		2 (15.3%)		1 (33.3)	4 (100%)
**Total** **N = 217**	36 (16.6%)	7 (3.22%)	8 (3.7%)	10 (4.61%)	20 (9.22%)	9 (4.1%)	6 (2.8%)	10 (4.61%)	8 (3.7%)	12 (5.53%)	5 (2.3%)	29 (13.4%)	21 (9.7%)	14 (6.45%)	13 (5.9%)	2 (0.9%)	3 (1.4%)	4 (1.8%)

Abbreviations: AVM: Arterio-Venous Malformation; VM: Venous Malformation; VLM: Venous-Lymphatic Malformation; LM: Lymphatic Malformation; Others: unconfirmed Malformation; No data: incomplete data.

**Table 3:** Overview of the minimally invasive therapy variants stratified by the type of vascular malformation and sex.

Type of Therapy		No Therapy	Therapy with Unknown Agents	Electro-Sclerotherapy	Embolization	Embolization + Surgery	Embolization + Cemento-Plasty	Sclero- therapy	Sclerotherapy + Surgery	Sclerotherapy + Electro-Sclerotherapy	Sclerotherapy + Embolization	Sclerotherapy + Cementoplasty	Sclerotherapy, Embolization + Surgery	Cemento- Plasty
**Sex**	**Type of VMF**													
**Male ** **N = 78**	AVM	4	1	0	1	0	0	3	0	0	2	0	2	0
	VM	18		0	3	0	0	10	0	1	2	1	0	0
	VLM	0		0	0	0	0	4	1	0	0	0	0	0
	LM	3		0	0	0	0	2	0	0	0	0	0	0
	Others	3	1	0	2	0	0	1	0	0	1	0	0	1
	N/A	10		1	0	0	0	0	0	0	0	0	0	0
	Total	38 (35.2%)	2 (50%)	1 (50%)	6 (28.6%)	0	0	20 (31.7%)	1 (100%)	1 (100%)	5 (50%)	1 (100%)	2 (100%)	1 (50%)
**Female** **N = 134**	AVM	9		0	8	0	0	9	0	0	5	0	0	0
	VM	31	1	1	5	0	0	29	0	0	0	0	0	0
	VLM	3		0	1	0	0	3	0	0	0	0	0	0
	LM	2		0	0	0	0	1	0	0	0	0	0	0
	Others	5	1	0	0	1	1	0	0	0	0	0	0	
	N/A	15		0	1	0	0	1	0	0	0	0	0	1
	Total	65 (60.2%)	2 (50%)	1 (50%)	15 (71.4%)	1 (100%)	1 (100%)	43 (68.3%)	0	0	5 (50%)	0	0	1 (50%)
**T** **N = 5**	N/A	5	0	0	0	0	0	0	0	0	0	0	0	0
	Total	5 (4.6%)	0	0	0	0	0	0	0	0	0	0	0	0
**Total** **N = 217**		108 (49.8%)	4 (1.8%)	2 (0.9%)	21 (9.7%)	1 (0.5%)	1 (0.5%)	63 (29%)	1 (0.5%)	1 (0.5%)	10 (4.5%)	1 (0.5%)	2 (0.9%)	2 (0.9%)

Abbreviations: AVM: Arteriovenous Malformation; LM: Lymphatic Malformation; VLM: Venous-lymphatic Malformation; VM: Venous Malformation; Others: unconfirmed Malformation; N/A: incomplete data.

**Table 4:** Changes in MSI and lesion volume on MRI from baseline to follow-up 1 and follow-up 2 after sclerotherapy.

Type of VMF	MRI Parameter	MRI Sequence	Baseline	Follow-Up 1	Follow-Up 2	*p*-Value
**AVM**	MSI	T1	404.08	397.44	370.67	0.983 (BL vs. FU1)/0.819 (FU1 vs. FU 2)
		T2	671.92	586.11	470.17	0.834 (BL vs. FU1)/0.120 (FU1 vs. FU 2)
		ceT1	698.75	632.43	752.2	0.156 (BL vs. FU1)/0.863 (FU1 vs. FU 2)
	MSI ratio	T1	1.23	1.22	1.17	0.575 (BL vs. FU1)/0.133 (FU1 vs. FU 2)
		T2	3.58	3.75	3.63	0.855 (BL vs. FU1)/0.409 (FU1 vs. FU 2)
		ceT1	1.69	2.15	1.93	0.570 (BL vs. FU1)/0.466 (FU1 vs. FU 2)
	Volume in cm^3^		59.5	67.2	58.16	0.557 (BL vs. FU1)/0.180 (FU1 vs. FU 2)
**VM**	MSI	T1	689.11	493.86	671.57	0.231 (BL vs. FU1)/0.231 (FU1 vs. FU 2)
		T2	1007.9	465.26	653.2	**0.028 (BL vs. FU1)**/0.466 (FU1 vs. FU 2)
		ceT1	832.2	589.1	942.25	0.797 (BL vs. FU1)/0.279 (FU1 vs. FU 2)
	MSI ratio	T1	1.41	1.08	0.81	0.289 (BL vs. FU1)/0.224 (FU1 vs. FU 2)
		T2	4.24	3.77	3.60	0.729 (BL vs. FU1)/0.951 (FU1 vs. FU 2)
		ceT1	3.40	2.50	3.16	**0.011 (BL vs. FU1)**/0.216 (FU1 vs. FU 2)
	Volume in cm^3^		119.9	100.54	108.24	0.866 (BL vs. FU1)/0.733 (FU1 vs. FU 2)
**VLM**	MSI	T1	740.25	441.8	471.5	0.477 (BL vs. FU1)/0.166 (FU1 vs. FU 2)
		T2	1330.2	459	542	0.309 (BL vs. FU1)/0.970 (FU1 vs. FU 2)
		ceT1	1055	441.8	563	0.300 (BL vs. FU1)
	MSI ratio	T1	0.86	0.99	0.91	0.980 (BL vs. FU1)/0.860 (FU1 vs. FU 2)
		T2	3.73	3.55	2.48	0.555 (BL vs. FU1)/0.536 (FU1 vs. FU 2)
		ceT1	1.69	1.46	1.04	0.999 (BL vs. FU1)
	Volume in cm^3^		78.9	62.32	71.7	0.271 (BL vs. FU1)/0.056 (FU1 vs. FU 2)
**LM**	MSI	T1	320	439.33	-	0.560 (BL vs. FU1)
		T2	1184.7	676.33	-	0.208 (BL vs. FU1)
		ceT1	557.33	285.33	-	**0.029 (BL vs. FU1)**
	MSI ratio	T1	1.18	1.65	-	0.236 (BL vs. FU1)
		T2	4.22	5.13	-	0.233 (BL vs. FU1)
		ceT1	1.19	1.56	-	0.647 (BL vs. FU1)
	Volume in cm^3^		270.25	376.15	-	0.449 (BL vs. FU1)

Note: Due to the limited number of cases, no additional statistics were calculated for other split data (i.e., lymphatic malformations, venous-lymphatic malformations). Abbreviations: VMF: vascular malformation; MSI: Mean Signal Intensity; ce: Contrast enhanced; AVM: Arteriovenous Malformation; VM: Venous Malformation. Bold: Highlights the significant results.

**Table 5:** Changes in MSI and lesion volume on MRI from baseline to follow-up 1 and follow-up 2 after embolization.

Type of VMF	MRI Parameter	MRI Sequence	Baseline	Follow-Up 1	Follow-Up 2	*p*-Value
**AVM**	MSI	T1	327.44	402.5	371.5	0.93 (BL vs. FU1)
		T2	324.22	444.33	508	0.53 (BL vs FU1)/0.667 (FU1 vs. FU2)
		ceT1	495.22	458	317	0.365 (BL vs FU1)/0.616 (FU1 vs. FU2)
	MSI ratio	T1	1.11	1.03	0.91	0.376 (BL vs. FU1)
		T2	3.71	2.6	3.47	0.318 (BL vs FU1)/0.588 (FU1 vs. FU2)
		ceT1	1.64	1.66	1.29	0.457 (BL vs FU1)/0.901 (FU1 vs. FU2)
	Volume in cm^3^		180.43	164.23	79.21	0.135 (BL vs. FU1)/0.5 (FU1 vs. FU2)
**VM**	MSI	T1	397.6	293.75	-	0.768 (BL vs. FU1)
		T2	858.13	348.75	-	0.424 (BL vs. FU1)
		ceT1	416.17	428.33	-	0.971 (BL vs. FU1)
	MSI ratio	T1	0.94	1.17	-	0.526 (BL vs. FU1)
		T2	5.21	2.87	-	0.419 (BL vs. FU1)
		ceT1	1.69	2.53	-	0.068 (BL vs. FU1)
	Volume in cm^3^		13.86	29.2	-	0.445 (BL vs. FU1)

Note: No statistics were calculated for one or more split data. (Too little available/comparable data). Significant differences in MSI are highlighted in bold and grey. Abbreviations: VMF: vascular malformation; MSI: Mean Signal Intensity; BL: Baseline; FU1: Follow-Up1; FU2: Follow-Up 2; ce: Contrast enhanced; AVM: Arteriovenous Malformation; VM: Venous Malformation.

## Data Availability

Data will be shared by the corresponding author upon reasonable request.

## Supplementary Materials

The following supporting information can be downloaded at: https://www.mdpi.com/article/10.3390/life14101270/s1. Figure S1. Representative MRI scans of untreated high-flow arteriovenous (AVM) and low-flow lymphatic (LM) and venous malformations (VM). (A,B) depict a high-flow AVM in the groin and gluteal muscles on coronal contrast-enhanced T1-weighted sequence (A) and MR angiography (B). (C,D) show a low-flow LM in the pelvis with an enhanced cyst wall on contrast-enhanced T1-weighted (C) and hyperintensity with characteristic fluid-fluid-levels on T2-weighted MRI (D). (E,F) show a low-flow VM in the ventral lower leg, which is isointense to the surrounding muscle on axial T1-weighted MRI (E) and hyperintense on axial T2-weighted sequences (F). Table S1. MRI characteristics of vascular malformations adapted from Compendium Vascular Anomalies. Table S2. Most frequently used active agents and treatment regimens for minimally invasive image-guided therapy of vascular malformations. Table S3. Symptoms before and after minimally invasive image-guided therapy.
