# Supplementary material for: Regimens and Response Assessment in Minimally Invasive Image-Guided Therapies for Vascular Malformations: Insights from a Large Cohort Study at a Tertiary-Care Hospital
Source: Life (Basel). 2024 Oct 5;14(10):1270. doi: 10.3390/life14101270 (PMC11508878; doi:10.3390/life14101270)
Supplement: Supplementary file 1 [file life-14-01270-s001.zip › Figure S1.pdf]

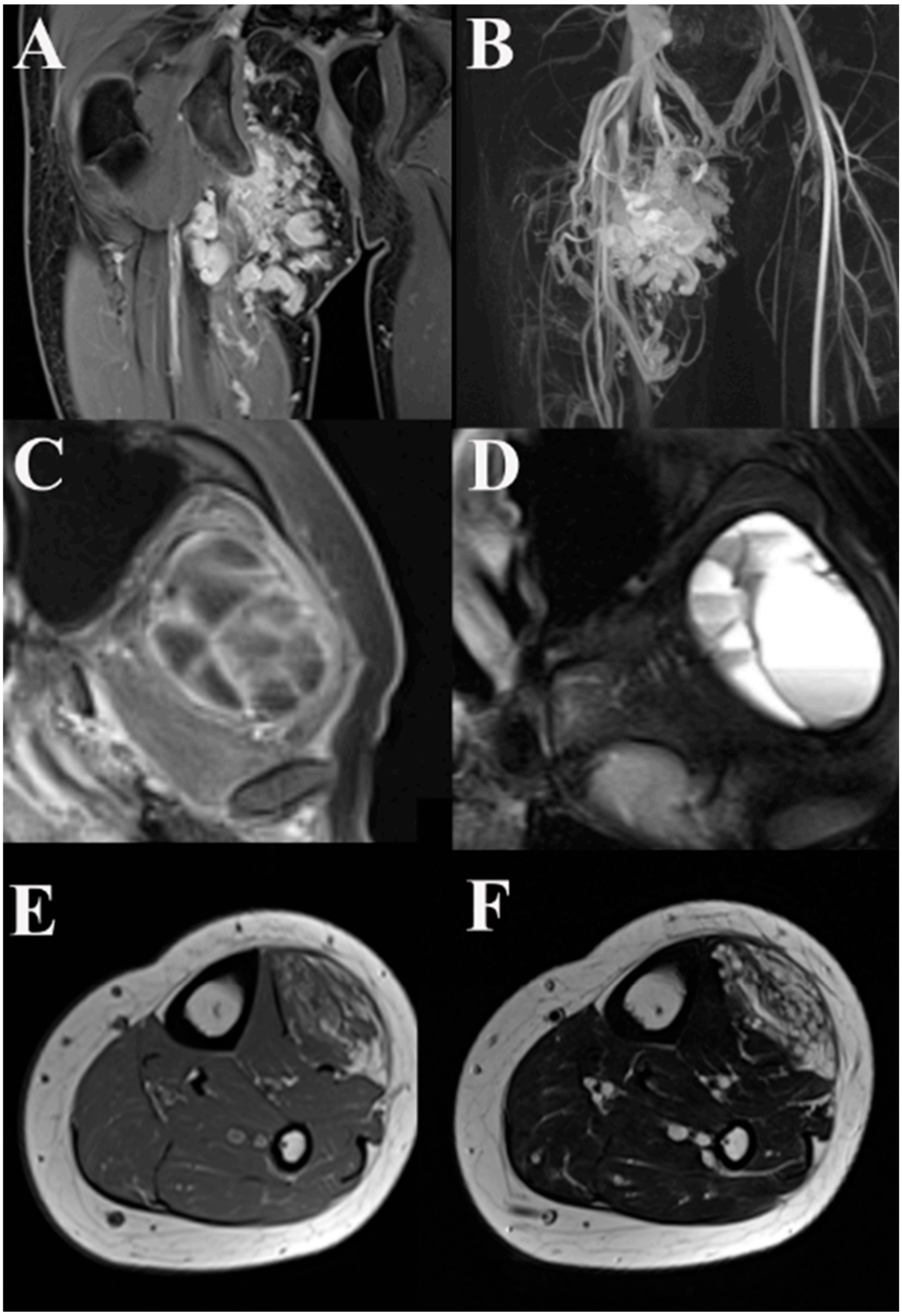

Fig 1 Representative MRI scans of untreated high-flow arteriovenous (AVM) and low-flow lymphatic (LM) and venous malformations (VM). **A** and **B** depict a high-flow AVM in the groin and gluteal muscles on coronal contrast-enhanced T1-weighted sequence (A) and MR angiography (B). **C** and **D** show a low-flow LM in the pelvis with an enhanced cyst wall on contrast-enhanced T1-weighted (C) and hyperintensity with characteristic fluid-fluid-levels on T2-weighted MRI (D**)**. **E** and **F** show a low-flow VM in the ventral lower leg, which is isointense to the surrounding muscle on axial T1-weighted MRI (E) and hyperintense on axial T2-weighted sequences (F).
