# Supplementary material for: Regimens and Response Assessment in Minimally Invasive Image-Guided Therapies for Vascular Malformations: Insights from a Large Cohort Study at a Tertiary-Care Hospital
Source: Life (Basel). 2024 Oct 5;14(10):1270. doi: 10.3390/life14101270 (PMC11508878; doi:10.3390/life14101270)
Supplement: Supplementary file 1 [file life-14-01270-s001.zip › Table S1.pdf]

Table S1 MRI characteristics of vascular malformations adapted from *Compendium Vascular Anomalies* [15].

Type of VMF	Description	T1-sequence	T2-sequence	Twist-Angio	Contrast-enhanced T1-sequence
**AVM**	Nidus, dilated vessels, flow-related aneurysms	Nidus nearly invisible, dilated vessels, flow-related aneurysms, flow voids	Nidus nearly invisible, dilated vessels, flow-related aneurysms, flow voids	Nidus immediately contrasted, draining veins immediately contrasted, Flow-related aneurysms	Dilated vessels to and from MF, Flow-related aneurysms
**VM**	Phlebolites & thrombi, septated, lobulated mass with low space-occupying effect	Isointense (to muscle), fluid-fluid-levels in large VM	Hyperintense, fluid-fluid-levels in large VM	None to very slow contrast agent pooling	Homogenous enhancement
**LM**	Cystic lesion	Iso- to hypointense, fluid-fluid-levels	Hyperintense, fluid-fluid-levels	Not visible	Only enhancement of cystwall

Abbreviations: LM**:** Lymphatic Malformation VM**:** Venous Malformation AVM**:** Arteriovenous Malformation VMF**:** Vascular Malformation
