# Supplementary material for: Regimens and Response Assessment in Minimally Invasive Image-Guided Therapies for Vascular Malformations: Insights from a Large Cohort Study at a Tertiary-Care Hospital
Source: Life (Basel). 2024 Oct 5;14(10):1270. doi: 10.3390/life14101270 (PMC11508878; doi:10.3390/life14101270)
Supplement: Supplementary file 1 [file life-14-01270-s001.zip › Table S2.pdf]

Table S2 Most frequently used active agents and treatment regimens for minimally invasive image-guided therapy of vascular malformations.

Type	Category	Trade name	Active ingredient	Indication	Combinable with
**Sclerosant**	Alcohol based	Braun ethanol 95%	Ethanol 95%	VM	Many sclerosing agents to increase effect
		Sklerogel	Ethanol 96%	VM	if nec. Lipiodol
		Discogel	Ethanol (high-percentage)	VM	if nec. Lipiodol
	Other	Ethoxysclerol	Lauromacrogol	VM	
		Fibrovein	Natriumtetradecylsulfate	VM	CA
		Various	Doxycyclin	LM	CA, Lipiodol
		Various	Bleomycin	VM, LM	e.g. Electroporation, CA, Lipiodol
		Picibanil	Ok-432	LM	CA, lipiodol
**Glue**	Adhesive	Histoacryl	N-butylcyanoacrylate	All	All mixed with lipiodol, preinjection of glucose to avoid premature *polymerization*
		Magic glue	Cyanoacrylate	All	
		Glubran	N-butylcyanoacrylate	All	
	Kohesive	Onyx	Ethylenalcohol-copolymer	AVM	
		Squid	Ethylenalcohol-copolymer	AVM	

Abbreviations: VM: Venous Malformation LM: Lymphatic Malformation AVM: Arterio-vanous Malformation CA: Contrast Agent
