# Supplementary material for: Regimens and Response Assessment in Minimally Invasive Image-Guided Therapies for Vascular Malformations: Insights from a Large Cohort Study at a Tertiary-Care Hospital
Source: Life (Basel). 2024 Oct 5;14(10):1270. doi: 10.3390/life14101270 (PMC11508878; doi:10.3390/life14101270)
Supplement: Supplementary file 1 [file life-14-01270-s001.zip › Table S3.pdf]

Table S3 Symptoms before and after minimally invasive image-guided therapy.

	**Pain**	**Pressure**	**Function loss**	**Spontaneaous bleeding**	**Ulceration**	**Asthetic limitations**	**Spread to neighboring tissue**	**Swelling**
Pre-therapy (n=156)	112 (71.8%)	36 (23.1%)	34 (21.8%)	6 (3.85%)	1 (0.64%)	3 (1.92%)	6 (3.85%)	72 (46.15%)
Post-therapy (n=20)	11 (55%)	2 (10%)	5 (25%)		1 (5%)			5 (25%)
	**Pulsation**	**Temperature differences**	**Cardiac issues**	**Skin Discoloration**	**Numbness**	**Necrosis**	**Cramps**	
Pre-therapy (n=156)	7 (4.5%)	6 (3.85%)	2 (1.28%)	9 (5.8%)	1 (0.64%)			
Post-therapy (n=20)				1 (5%)	2 (10%)	3 (15%)	1 (5%)	
